# Leaching Mechanism and Health Risk Assessment of As and Sb in Tailings of Typical Antimony Mines: A Case Study in Yunnan and Guizhou Province, Southwest China

**DOI:** 10.3390/toxics10120777

**Published:** 2022-12-12

**Authors:** Ziyou Bai, Yinping He, Zhiwei Han, Fuzhong Wu

**Affiliations:** 1 Resource and Environmental Engineering College, Guizhou University, Guiyang 550025, China; 2 School of Materials and Metallurgy, Guizhou University, Guiyang 550025, China; 3 Key Laboratory of Karst Georesources and Environment, Ministry of Education, Guiyang 550025, China

**Keywords:** mining tailings, leachate, chemical Sb and As species, scanning electron microscope, health risk assessment

## Abstract

The weathering and leaching of mining tailings have released large amounts of antimony (Sb) and arsenic (As), causing serious pollution in the surrounding soil, water, and sediments. To understand the leaching characteristics of Sb and As in mining tailings, Zuoxiguo and Qinglong mining tailings were collected for analysis. The average content of Sb in Zuoxiguo and Qinglong tailings was 5902.77 mg/kg and 1426.43 mg/kg, respectively, while that of As was 412.53 mg/kg and 405.26 mg/kg, respectively, which exceeded the local background value. Furthermore, the concentrations of Sb in the leachate of Zuoxiguo and Qinglong increased with time; the average Sb concentration in the leachate of Zuoxiguo and Qinglong was 1470.48 μg/L and 70.20 μg/L, respectively, while that of the As concentration was 31.20 μg/L and 6.45 μg/L, respectively. This suggests that the concentrations of Sb and As in the leachate of Zuoxiguo are both higher than those in the leachate of Qinglong and that the pH of the leachate of Zuoxiguo and Qinglong significantly changed within the first day under different initial pH conditions, and tended to be between 6 and 8, after one day. The results of the average health risk index showed that As in the leachate from Zuoxiguo and Qinglong for children was 5.67 × 10^−4^ and 9.13 × 10^−5^, respectively, and 4.43 × 10^−4^ and 7.16 × 10^−5^, respectively, for adults. As in the leachate from Zuoxiguo poses serious carcinogenic risks for residents, and in the study area, As poses a serious threat to human health. Therefore, the local government must manage As in these areas.

## 1. Introduction

Mining tailings are one of the most important contributors to heavy metal pollution [1]. Indeed, mining activities increase the levels of heavy metals in agricultural soils, surface water, groundwater, and plants in surrounding areas [2,3,4] and pose significant health risks to residents and miners [5,6]. Mineral resources dominate China’s energy consumption [7]; however, despite China’s development has gained economic benefits, heavy metal pollution has increased [8]. Heavy metals in mining tailings can be transferred through weathering and rainfall to the surrounding soils and water bodies [9]. Liquids are a key medium for the migration of heavy metals from tailings [10], which can be leached by rainfall and subsequently migrate with surface runoff or leachate before being absorbed by the mechanistic components of soils and sediments [11]. Heavy metal pollution from mining activities, tailing weathering, and leaching is a significant challenge in southwest China [12]. The reported levels of antimony (Sb) and arsenic (As) in soil, water bodies, and vegetation around the Xikuangshan mine, an active antimony mine located in southwest China, exceed those of local background levels [13,14,15], which can adversely affect the surrounding environment and residents.

Sb is a heavy metal with a density of >5 g/cm^3^ [16], while As is categorized as a heavy metal [17]. As and Sb pollution in the natural environment is persistent, non-degradable, and irreversible [18], which has become a major concern [19,20], especially in antimony mining areas, where As and Sb pose a significant health risk to residents and animals [21]. Sb and As contents in hair samples from residents near antimony mine were observably higher compared with those in non-mining areas, suggesting that the Sb and As released from Qinglong mining areas adversely affect human health [22]. Sb has attracted significant attention due to its toxicity and environmental pollution [23]. However, along with As, data on Sb environmental and health risks are limited compared with other heavy metals such as Pb, Zn, Cd, and Cu [24]. 

The study of the leaching characteristics of heavy metals in tailings can provide insights into their impact on groundwater and soil [25,26]. Previous studies have identified the risks associated with the leaching of heavy metals in tailings on the soil environment [27]. Moreover, the release of heavy metals in tailings is not only influenced by the total concentration of heavy metals and the pH of tailings but also the chemical species of heavy metals [27,28]. In addition, the leaching of heavy metals under different environmental conditions can be simulated [29]; hence, their threat to the ecology in the area and surrounding populations can be assessed. 

Therefore, in this study, Zuoxiguo and Qinglong antimony mines were selected in southwest China as the study areas to: (1) investigate the total content of As and Sb in the tailings and tailings components; (2) study the leaching characteristics of As and Sb in the tailings based on the concentration of As and Sb in the leachate and the chemical species of As and Sb in the tailings under different initial pH conditions; and (3) derive the health risks following human consumption of As and Sb in the leachate and provide suggestions to policy makers to mitigate such risks.

## 2. Materials and Methods

### 2.1. Study Area

Qinglong antimony mine (approximately 25°33′—26°11′ N, 105°01′—105°25′ E), located in Dachang Town, Qinglong County, Guizhou Province, has an average annual temperature of 17 °C and precipitation of approximately 1500 mm, as the area hosts a subtropical monsoon climate. Zuoxiguo antimony mine (approximately 23°22′—23°41′ N, 103°31′—103°57′ E), located in Zuoxiguo Village, Beige Town, Kaiyuan City, Yunnan Province, hosts a subtropical plateau monsoon climate, with distinct dry and wet seasons. The area has an annual average temperature of 24.2 °C and rainfall of 1450 mm. The mining history of these two typical Sb mines is extensive [18], posing a serious heavy metal pollution challenge. Qinglong and Zuoxiguo antimony mines have terminated production for many years [30,31].

### 2.2. Sampling and Sample Preparation

**Figure 1:**
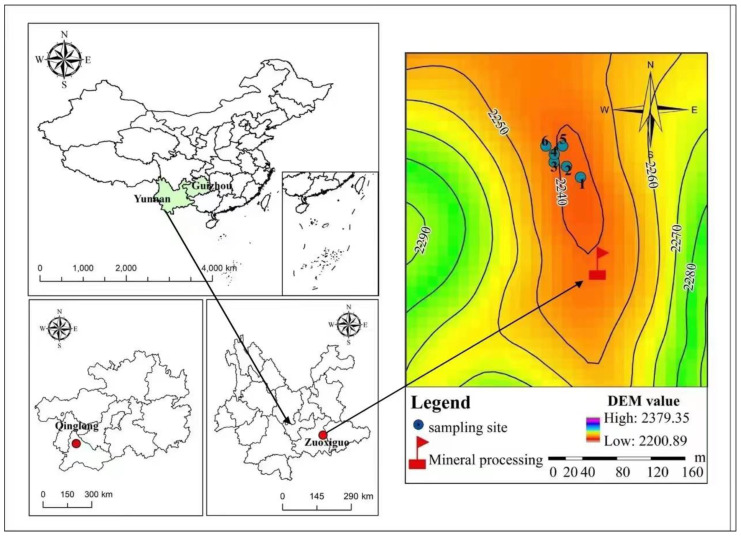
Location of the two typical antimony mines.

According to the different landforms of the two antimony mines, the sampling layout in the two tailings were also different. After removing the sundries horizons from the sampling sites, samples were drilled via the method of mixed sampling. The landform of the Zuoxiguo antimony mine is an open-type, and heavy metals could easily migrate along the direction of water flow; therefore, six 30 cm samples were collected from high to low using a stainless-steel shovel in each sample square via the double diagonal five-point mixing method. In contrast, the Qinglong antimony mine is surrounded by mountains, while the middle is a basin where heavy metals are more likely to migrate and diffuse downwards and are less likely to migrate over long distances. A total of one tailing sample was collected (Figure 1). Each sample was marked with a marker pen, and the location of every sampling site was recorded using the global positioning system (GPS). All samples were freeze-dried for 5–7 days to prevent oxidation. Once dried, part of each sample was ground with an agate mortar by hand, and the powdered samples were sieved through a <10-mesh sieve for sand property determination and <200-mesh sieve for element analysis. Tailing sample pieces (0.05 g) were accurately weighed and dissolved in a 3 mL acid mixture with a 2:1 volume ratio of concentrated HNO_3_ and concentrated HF to analyse the total content of heavy metals. Sb and As concentrations in the extracts were analysed using an atomic fluorescence spectrometer (AFS-8510, China).

### 2.3. Sequential Extraction and Leaching Test

Chemical species of tailings were extracted using improved sequential extraction (BCR). Briefly, the acid-soluble fraction (F1) was extracted using 40 mL 0.11 mol·L^−1^ CH_3_COOH; then, the reducible fraction (F2) was extracted with 40.00 mL 0.50 mol·L^−1^ hydroxylamine hydrochloride (NH_2_OH·HCl), while the oxidizable fraction (F3) was digested with 10.00 mL 8.80 mol·L^−1^ NH_2_OH·HCl and extracted with 50.00 mL 1.00 mol·L^−1^ ammonium acetate (CH_3_COONH_4_). Sb and As concentrations in F1, F2, and F3 were measured using an atomic fluorescence spectrometer (AFS-8510, China). The residual fraction (F4) was calculated as the difference between the concentration of the sum of the three fractions (F1 + F2 + F3) and the total Sb and As concentrations [32].

The pH-static leaching experiments were performed for 9 days. Tailing samples (2 ± 0.01 g) were placed in a 50 mL polyethylene bottle, and 40 mL of prepared extraction was added. Separate bottles were used for each sampling to ensure identical ratios. The pH values of 3, 5, 6.87, and 9 were to account for acidic, neutral, and basic conditions encountered in the mining areas. Sulphuric acid and sodium hydroxide were added to adjust the pH. The reactor was placed in a gas bath thermostatic shaker at 120 r/min. The leachate was sampled after days 1, 3, 5, 7, and 9 and filtered through a 0.45 μm membrane. A PHS-3c pH meter was used to continuously measure the tailing supernatant three times, and the average value was the leachate used for the analysis of Sb and As concentrations.

On the basis of the previously established standard for determining heavy metals in soil (HJ680-2013), a total of 6 samples to be analysed were inserted using a blank sample, in accordance with the 10% standard. During analysis, procedural gaps, parallel experiments, and soil samples (obtained as per national standard GSS-4) were considered for quality control. The calibration curves of Sb and As standard solutions showed determination coefficients exceeding 0.999 with recovery rates between 80.0 and 105.0%. Ultrapure water was used as the experimental water, while all reagents utilized were guaranteed reagents. The container was soaked in 10% HNO_3_ solution for more than 24 h, rinsed with ultra-pure water, and dried prior to use. All experimental equipment was dipped in nitric acid (12%) for more than 24 h and washed with deionized water at least three times. Arcmap10.6, Origin 2021, and Microsoft Excel 2017 were used for data processing and graphics drawing.

### 2.4. Health Risk Assessment

The human health risk assessment was divided into carcinogenic and non-carcinogenic assessments. As is a carcinogen via the drinking route, while Sb is a non-carcinogen. In this study, health risk was considered to the population through the drinking water route when leachate containing As and Sb contaminates groundwater. Therefore, the carcinogenic risk of As and the non-carcinogenic risk of Sb were analysed separately.

#### 2.4.1. Carcinogenic Risk Model

In general, even a very small number of carcinogenic risk substances can have negative affect on human health. The expression of the evaluation model is as follows:

(1) $$R_{c}=(D_{i}\times SF_{i})/70\;$$

where *R_c_* is the per capita carcinogenic risk generated by chemical carcinogens through the surface of water sources, a^−1^; *D_i_* is the average daily exposure dose per unit of toxic substances through the surface of drinking water sources, mg·(kg·d)^−1^; *SF_i_* is the carcinogenic coefficient of chemical carcinogens ingested through the surface of drinking water sources, mg·(kg·d)^−1^ [33].

#### 2.4.2. Non-Carcinogenic Risk Model

Non-carcinogens are only hazardous to human health if they exceed a threshold value. The non-carcinogenic risk is described by the commonly used risk index (HI) evaluated as follows:

(2) $$R_{n}=\frac{D_{i}}{RfD\times 70}\times 10^{-6}$$

where *R_n_* is the per capita annual health risk from non-carcinogenic substances exposed through drinking water sources, a^−1^; *D_i_* is the average daily exposure dose per body of toxic substances exposed through water sources, mg·(kg·d)^−1^; *RfD* is the carcinogenic coefficient of non-carcinogenic substances ingested through drinking water sources, mg·(kg·d)^−1^; 70 is the average life expectancy, in years [34].

The average daily exposure dose *Di* by the drinking water route is calculated separately for adults and children using the following formula:

(3) $$Adult:D_{i}=2.2C_{i}/64.3$$

(4) $$Child:D_{i}=\left(1.0C_{i}\right)/22.9$$

where *C_i_* is the concentration of the chemical carcinogen, mg/L; 2.2 is the average daily water intake of adults, L/d; 64.3 is the average body weight of adults, in kg; 1.0 is the average daily water intake of children, L/d; 22.9 is the average body weight of children, in kg [34].

The classification system is based on the International Agency for Research on Carcinogenesis (IARC) and the World Health Organization’s (WHO) comprehensive evaluation of the carcinogenicity of chemical substances, combined with the US EPA recommended values. Among the two toxic elements measured in this study, the carcinogenic intensity factor SF for As was 15 mg·(kg·d)^−1^ and the drinking water exposure reference dose *RfD* for Sb was 0.004 mg·(kg·d)^−1^. The health risk in the water environment was classified into five levels: *R_c_*/*R_n_* < 1.0 × 10^−6^ a^−1^, low risk; 1.0 × 10^−6^ a^−1^ ≤ *R_c_*/*R_n_* < 1.0 × 10^−5^ a^−1^, considerable; 1.0 × 10^−5^ a^−1^ ≤ *R_c_*/*R_n_* < 5.0 × 10^−5^ a^−1^, medium; 5.0 × 10^−5^ a^−1^ ≤ *R_c_*/*R_n_* < 1.0 × 10^−4^ a^−1^, high risk; *R_c_*/*R_n_* > 1.0 × 10^−4^ a^−1^, serious risk [35].

## 3. Results and Discussion

### 3.1. Mineral Composition and Toxic Elements Content in Tailings

**Figure 2:**
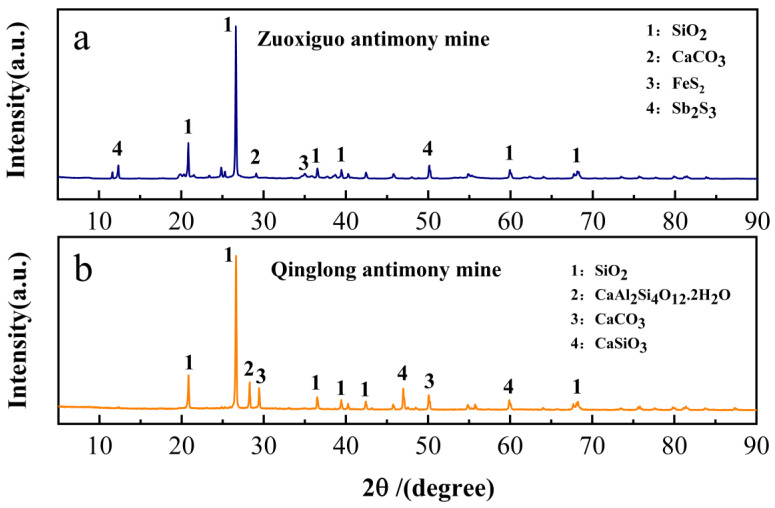
XRD pattern of two mining tailings. ((**a**): XRD analyse for Zuoxiguo mining tailings; (**b**): XRD analyse for Qionglong mining tailings).

The peak XRD analysis pattern intensities primarily represent the crystallinity of different phases; hence, the contents of different components in the samples cannot beanalyzed. The XRD patterns of the tailings showed different diffraction patterns (Figure 2). The compositions of the Zuoxiguo antimony mining tailings were SiO_2_, CaCO_3_, FeS_2_, and Sb_2_S_3_, a typical sulphide mining tailing. Conversely, the fractions of the Qinglong antimony mine tailings were SiO_2_, CaCO_3_, CaSiO_3_, and CaAl_2_Si_4_O_12_-2H_2_O. The primary component of the tailings of both antimony mines was SiO_2_. Compared with the Zuoxiguo antimony mine, the tailings of the Qinglong antimony mine contained less Sb (Table 1); therefore, no significant peaks in the Sb phase were detected. Yet, the oxides of secondary Sb, such as cubic Sb_2_O_3_ and rhombic Sb_2_O_3_, were the most important weathering of Sb_2_S_3_ products. The tailing sand contained SiO_2_ and CaCO_3_, which are associated with silicification and carbonatization in wall rock alteration, respectively.

The tailings of Zuoxiguo and Qinglong antimony mines contained large amounts of Sb and As (Table 1), with the contents of Sb and As in the Qinglong antimony mine being 637 and 20 times higher than the local soil background values, respectively. The average content of Sb and As in the Zuoxiguo mine was 771 and 27 times higher than the local soil background values, respectively. Notably, the pH of the Qinglong antimony mine tailings was much higher than that of the Zuoxiguo tailings, which is neutral to weakly alkaline. Meanwhile, the pH of the Zuoxiguo antimony mining tailings was acidic, which is more conducive to the migration and transformation of heavy metals under acidic conditions [36]. This may explain the significantly higher Sb in the Zuoxiguo antimony mining tailings than Qinglong antimony mining tailings. In addition, combined with the XRD in Figure 2, pyrite was identified in the tailings of the Zuoxiguo antimony mine. Under natural conditions, the pyrite in the tailings undergoes a redox reaction (2FeS_2_ + 7O_2_ + 2H_2_O→2Fe_2_ + 4SO_4_^2−^ + 4H^+^), thereby releasing a large number of hydrogen ions into the tailings [37], which lower the pH. Heavy metals associated with pyrite are released into the environment along with heavy metal carbonates [38], which may be another reason for the high Sb and As content in the tailings.

### 3.2. Leaching Characteristics of Different Antimony Mining Tailings

#### Leaching Characteristics of Sb and As in Tailings, and pH Changes of Leachate during Leaching

**Figure 3:**
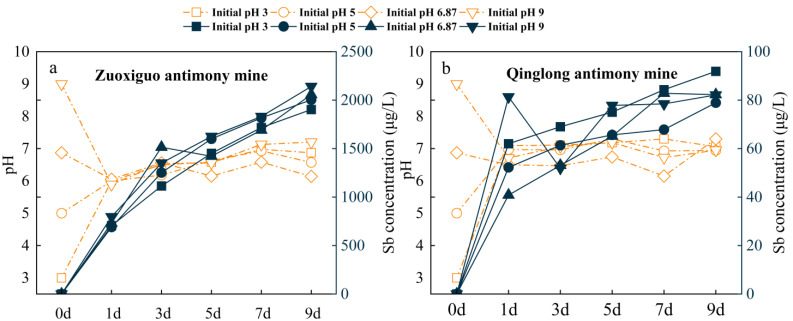
Leached concentration changes of Sb in different mines and pH changes of the leached solution (corresponding dashed labels). ((**a**): Sb’s leaching characteristics and pH changes in Zuoxiguo-tailings leachate; (**b**): Sb’s leaching characteristics and pH changes in Qinglong-tailings leachate).

Figure 3 shows the total Sb released from each mining tailing, which, although different, was increased with time, especially in Zuoxiguo mine tailings. The Sb release of the Zuoxiguo mine tailings was larger than the Qinglong mine tailings. 

Contrarily, the pH showed different trends, changing significantly within the first day regardless of the initial pH, and remained between 6 and 8, after one day, for days 2–9. Regardless of the pH at the initial condition, the release of Sb in the tailings of Zuoxiguo and Qinglong antimony mines was the highest within the first day, with an average release rate of 37%. This indicates that the artificially given pH value within the first day helped the Sb leaching process. Notably, the alkaline conditions were more favourable to Sb release than acidic conditions during the leaching process of the Zuoxiguo antimony mining tailings [39]. The antimony mining tailings consist of pyroxene, which can dissolve in water to form the hydroxide Sb(OH)_3_ [40], the expression of which is:

$${\mathrm{Sb}}_{2}S_{3}+6H_{2}O\;⟺\;2{\mathrm{Sb}}_{2}{\left(\mathrm{OH}\right)}_{3}+3H_{2}S$$

However, Sb(OH)_3_ behaves more like an acid than a hydroxide; therefore, it is often written as H_3_SbO_3_ (antimonous acid) and can dissociate to form an anion as follows:

$$H_{3}{\mathrm{SbO}}_{3}\;⟺\;H_{2}{\mathrm{SbO}}_{3}^{-}+H^{+}$$

As the pH increases, more H_3_SbO_3_ is converted to the anionic form; H_2_SbO_3_^−^ is very soluble in water [41,42]. Sb in the tailings of the Qinglong antimony mine was more readily released at an initial pH of 3. The trend of release under other pH conditions was consistent with that of Zuoxiguo. However, the Sb release in the tailings of Zuoxiguo was higher than that of Qinglong regardless of the initial pH conditions since the Sb content of the tailings of Zuoxiguo antimony mine was higher than that of Qinglong. The presence of FeS_2_ helped to facilitate Sb release and increased th4 Sb_2_S_3_ oxidation rate; therefore, in systems with more FeS_2_ input, Sb accumulation is increased [43]. Herein, the interaction between FeS_2_ and Sb_2_S_3_ increased Sb dissolution from Sb_2_S_3_. FeS_2_ has a higher resting potential than Sb_2_S_3_, 0.66 V vs. 0.12 V, respectively [44], which may stimulate Sb_2_S_3_ to release large amounts of Sb. Therefore, the Sb concentration in the Zuoxiguo leachate was higher than Qinglong.

**Figure 4:**
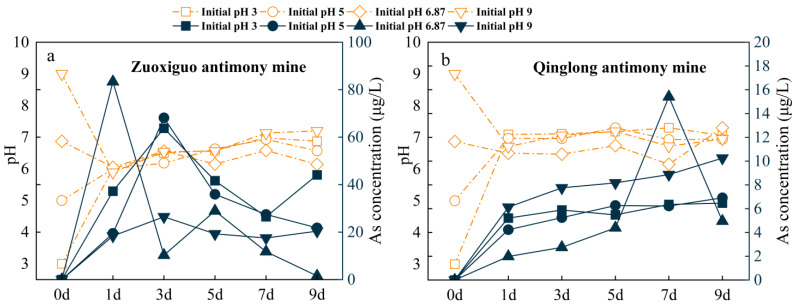
Leached concentration changes of As in Zuoxiguo and Qinglong mines and pH changes of the leached solution (corresponding dashed labels). ((**a**): As’s leaching characteristics and pH changes in Zuoxiguo-tailings leachate; (**b**): As’s leaching characteristics and pH changes in Qinglong-tailings leachate).

Conversely, the As release differed between the Zuoxiguo mining tailings and that of Qinglong mining tailings (Figure 4). The release of As from the Zuoxiguo mine tailings was much higher, while As concentration in the Zuoxiguo leachate increased with time before day 3, then decreased. Likewise, the pH showed different trends, varying significantly within the first day regardless of the initial conditions and remained between 6 and 8, after one day, for days 2–9. 

This suggests that the CaCO_3_ and other related components in the samples provided sufficient acid neutralization and buffering capacity. The release of As in the leachates from Zuoxiguo and Qinglong was significantly lower than that of Sb, possibly due to the source of As being toxic sand, which decomposed more slowly than pyroxene. This may be attributed to the formation of oxidation edges on the toxic sand particles consisting of Fe, As, S, Sb, and Ca that slowed down dissolution, which was not observed on the pyroxene [45]. In addition, the S content in the tailings of the Zuoxiguo antimony mine ranged from 3.065 g/kg to 14.718 g/kg, while the sulphur and sulphate in the tailings supported the leaching of As. As is chemically similar to S; hence, it can replace elemental S in sulphides, resulting in sulphides with high levels of As, such as FeAsS [46]. Reduction zones, such as rivers, sediments, and mines, can form authigenic FeS_2_ in which some dissolved As is bound during the formation of these FeS_2_ minerals; during transformation, As is bound to FeS_2_ and released during FeS_2_ dissolution [47]. Compared with other initial pH values, the release of As in the leachate from Zuoxiguo and Qinglong varied when the initial pH was 6.87; the release of As in the leachate from Zuoxiguo began to decrease within 1–3 days, while the release of As in the leachate from Qinglong sharply increased within 5–7 days. This indicates that the leaching variation of As is greater under neutral conditions, and the elemental activity is higher and easily influenced by other factors [48].

### 3.3. Influence of Chemical Sb and As Species in Tailings on Leaching Mechanism

**Figure 5:**
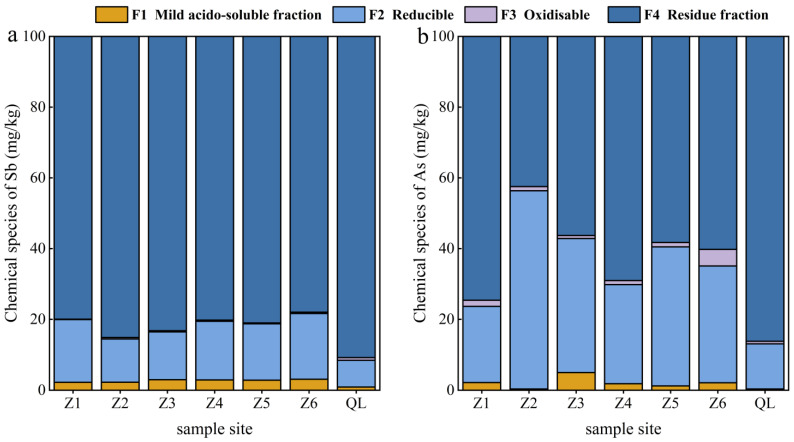
As and Sb chemical proportions at different points in tailings of Zuoxiguo and Qinglong.((**a**): Proportions of chemical Sb species in tailings of Zuoxiguo; (**b**): Proportions of chemical As species in tailings of Qinglong).

The improved BCR method was used to determine the Sb and As chemical species in the tailings of the Zuoxiguo and Qinglong antimony mines. The chemical species of Sb and As in the Qinglong mining tailings were predominantly F4 and F2, with overall proportions in the following order: F4 > F2 > F3 > F1. Qun et al. (2022) have reported that too high of a residue fraction indicated high contents of elements in the lattice, such as silicate, primary minerals, and secondary minerals. However, when the reducible was too high, elements bound with Fe and Mn hydrated oxides showed high content and activity, which are easily hydrolysed under pH < 7 with a certain bioavailability [49]. Figure 5 shows that F1 and F2 of Sb and As values in the Zuoxiguo mining tailings were higher than those of the Qinglong mining tailings, indicating that Sb in the Zuoxiguo mining tailings had stronger mobility and bioavailability. 

Hence, the release of Sb and As from the tailing sand in the leaching experiment of Zuoxiguo was higher than that of Sb and As from the tailing sand of Qinglong. Meanwhile, the F4 of Sb and As in the Qinglong mining tailings was higher than that of As in the Zuoxiguo mining tailings. The tailing sand of Qinglong had a higher binding degree with Sb and As elements, thereby hindering migration and transformation; hence, the release of Sb and As in the tailing sand of the Qinglong leaching experiment was significantly lower than that of Zuoxiguo.

### 3.4. Surface Changing of Tailings during Leaching

**Figure 6:**
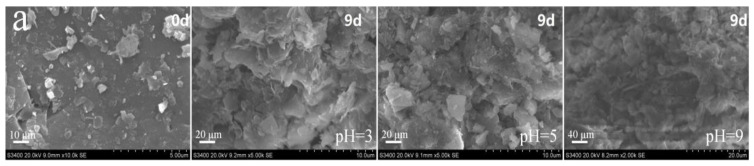

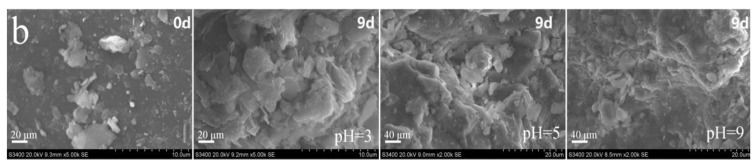
Tailing surface morphology changes before and after leaching: (**a**) Zuoxiguo antimony mine and (**b**) Qinglong antimony mine. Scale bar: 10 μm and 20 μm.

The surface shape of the tailings from the scanning electron microscope (SEM) is shown in Figure 6. The roughness of the tailing surfaces increased post leaching. The originally smooth surface of the tailings was eroded, while small holes appeared on the surface after 9 days of the alkaline initial leaching condition, and raised layers appeared on the surface after 9 days of the acid initial leaching condition (Figure 6). This illustrates that the dissolution of the tailings began with the tailing–water interface, while different initial conditions led to different tailings changes. However, it is possible that the reaction mechanism at the tailing–water interface is different, and so is the production under different initial pH conditions during leaching.

### 3.5. Health Risk Assessment of Leachate

During rainfall, the tailings’ leachate flows into the river from high to low along the terrain in the study area, which has exacerbated the negative health effects of Sb and As; therefore, a health risk assessment of Sb and As in the leachate was conducted. The highest leaching amount was used to calculate the health risk index under different pH conditions (Table 2). The non-carcinogenic and carcinogenic risks of Sb and As in the leachate at different pH were higher for children than adults. Children were at higher risk from As, while the health risk index of As in the leaching solution of Zuoxiguo was higher than that of Qinglong; hence, contaminated drinking water must be more strictly managed. In Table 2, the carcinogen element As in the leachate of the Zuoxiguo antimony mining tailings for the adult and children health risk index was higher than 1 × 10^−4^, a serious risk level, while the health risk index of Sb was less than 1.0 × 10^−6^, a low-risk level. The health risk index of carcinogenic As in the tailing leachate of the Qinglong antimony mine was greater than 1 × 10^−4^ for both adults and children at pH 6.87, a serious risk level. At pH 3, the health risk index for adults ranged from 1.0 × 10^−5^ to 5.0 × 10^−5^, a medium-risk level, while at pH 5 and 9, the health risk index for adults ranged from 5.0 × 10^−5^ to 1.0 × 10^−4^ at pH 5 and 9, a high-risk level. The health risk index for children was between 5.0 × 10^−5^ to 1.0 × 10^−4^ at pH 3, 5, and 9, a high-risk level; As had the highest health risk index for adults and children at pH 6.87, consistent with Zuoxiguo. On the contrary, the health risk indices of Sb were less than 1.0 × 10^−6^, a low-risk level. Therefore, the non-carcinogenic risk of Sb in the leachate is low-risk for both adults and children, i.e., within the acceptable range, while the carcinogenic risk of As exceeds a certain value, where As in the leachate of Zuoxiguo is a serious risk for both adults and children, which may be associated with the different toxicity of different heavy metals [49,50]. Therefore, it is necessary to introduce health risk assessment into water quality monitoring and evaluation around the study area and develop and implement appropriate contaminant control strategies.

## 4. Conclusions

In this study, Zuoxiguo and Qinglong antimony mining tailings were collected and analysed. The total contents of As and Sb in Zuoxiguo and Qinglong tailings, as well as the leaching mechanism of As and Sb, were determined, while the health risk evaluation of As and Sb in the leachate was conducted. The results showed that: (1) SiO_2_ was the main component of the Zuoxiguo and Qinglong antimony mining tailings; notably, Sb_2_S_3_ and FeS_2_ were identified in the tailings of the Zuoxiguo antimony mine. The average content of Sb in the Zuoxiguo and Qinglong tailings was 5902.77 mg/kg and 1426.43 mg/kg, respectively, while that of As was 412.53 mg/kg and 405.26 mg/kg, respectively, which were higher than the background values. (2) The average Sb concentration in the leachate of Zuoxiguo and Qinglong was 1470.48 μg/L and 70.20 μg/L, respectively, while that of As concentration in the leachate of Zuoxiguo and Qinglong was 31.20 μg/L and 6.45 μg/L, respectively. The release of Sb in the leachate of the Zuoxiguo and Qinglong tailings increased with time; the alkaline conditions contributed to the release of Sb. The release of As in the leachate of Zuoxiguo was first increased, then decreased, while the release of As in the leachate of Qinglong showed an increasing trend, in which the changes in As release in the leachate of Zuoxiguo and Qinglong at pH 6.87 differed from those of As release under other pH conditions. (3) The health risk index showed that the average carcinogenic risk of As in the Zuoxiguo and Qinglong leachates for children was 5.67 × 10^−4^ and 9.13 × 10^−5^, respectively, and 4.43 × 10^−4^ and 7.16 × 10^−5^ for adults, respectively, suggesting that As has a significantly higher risk for children compared with adults. Moreover, the carcinogenic risk index of As in the leachate of Zuoxiguo was higher than that of Qinglong. In contrast, the average non-carcinogenic risk index of Sb in the leachate of Zuoxiguo and Qinglong for children was 3.16 × 10^−7^ and 1.31 × 10^−8^, respectively, and 2.48 × 10^−7^ and 1.02 × 10^−8^ for adults, respectively, which were below the minimum limitation and within the acceptable range.

## Figures and Tables

**Table 1:** Total heavy metals in the Zuoxiguo and Qinglong mining tailings (mg/kg).

Item	Sb	As	pH
QL	1426.43	405.26	7.38
Background value of soil in Guizhou	2.24	20.00	n.d
Z1	13,689.70	541.20	3.73
Z2	9663.30	771.50	3.05
Z3	4450.90	218.00	2.99
Z4	3004.42	600.50	3.62
Z5	2428.35	206.00	3.49
Z6	2179.92	138.00	4.26
Background value of soil in Yunnan	7.64	14.90	n.d

n.d means that there is no pH background value in Guizhou and Yunnan.

**Table 2:** Annual health risks caused by Sb and As in the leachate of Zuoxiguo and Qinglong (a^−1^).

Item		pH	Sb	As
			Rn	Rc
Zuoxiguo	Adult	3.00	2.32 × 10^−7^	4.67 × 10^−4^
		5.00	2.45 × 10^−7^	5.00 × 10^−4^
		6.87	2.51 × 10^−7^	6.11 × 10^−4^
		9.00	2.62 × 10^−7^	1.94 × 10^−4^
	Child	3.00	2.97 × 10^−7^	6.00 × 10^−4^
		5.00	3.12 × 10^−7^	6.38 × 10^−4^
		6.87	3.21 × 10^−7^	7.80 × 10^−4^
		9.00	3.34 × 10^−7^	2.48 × 10^−4^
Qinglong	Adult	3.00	1.12 × 10^−8^	4.74 × 10^−5^
		5.00	9.64 × 10^−9^	5.07 × 10^−5^
		6.87	1.01 × 10^−8^	1.13 × 10^−4^
		9.00	1.00 × 10^−8^	7.52 × 10^−5^
	Child	3.00	1.43 × 10^−8^	6.05 × 10^−5^
		5.00	1.23 × 10^−8^	6.47 × 10^−5^
		6.87	1.29 × 10^−8^	1.44 × 10^−4^
		9.00	1.28 × 10^−8^	9.59 × 10^−5^

## Data Availability

Reasonable data requests can be obtained by email at bzy823396@163.com.
